# Patient Privacy and Integrated Care: The Multidisciplinary Health Care Team

**DOI:** 10.5334/ijic.5591

**Published:** 2020-11-19

**Authors:** John Eastwood, Isis Maitland-Scott

**Affiliations:** 1 Sydney Local Health District, Camperdown, NSW, AU

**Keywords:** integrated care, privacy, health and social care, multidisciplinary care, legislation

## Abstract

This article explores legislative provisions in relation to patient privacy in the context of integrated health and social care and the development of multidisciplinary health care teams that include practitioners from private sector and government agencies in the health, education, child protection, family welfare, disability, aged-care, housing, local government and criminal justice sectors. The definition of a multidisciplinary health care team and the extent to which health information can be shared within the team is examined. Australian Commonwealth and State legislation provides for the sharing within a health care team of health information where that is for the primary purpose it was collected, and for a secondary purpose where that is directly related to the primary purpose, or might be reasonably expected by the patient for the provision of their care. For this purpose consent is not required.

## Context

Information sharing among agencies and practitioners is essential for the provision of high quality health and social care. Cross-agency sharing of de-identified personal information is usually for advancing a shared understanding of population health and wellbeing and for improving the delivery of health and social services. Despite this common purpose the sharing of de-identified information can be difficult in some countries. Privacy concepts have a long historical tradition and can be traced back, for example, in English common law. Today they appear in the Universal Declaration of Human Rights and Article 8 of the European Convention on Human Rights. Each country and statutory jurisdiction will have different statutory provisions. The purpose here is to examine the situation in Australia with a particular focus on the State of New South Wales.

Health practitioners have a long tradition of maintaining the privacy of information obtained from patient consultations. Forrester and Griffiths observe that ‘there is an expectation that health professionals will keep confidential all information acquired as part of their role in the healthcare team. [1] With this expectation, patients feel confident to confide private information; ensuring the best care can be provided. This raises the question, who is the healthcare team, and to what extent can information be shared, used or disclosed within that team without the patient’s direct consent?

It is generally understood that it is common practice for patient confidential information to be shared within health care teams. A patient might reasonably expect that their physician’s attending nurse, and perhaps clerical staff, will have access to clinical notes taken during the consultation to pursue the best quality health care. Hospital health professionals are generally employed by the same agency, the same is often not the case for health care provided in community settings, making the sharing of information a more complex issue. It may not be immediately obvious to patients as to why personal information is shared within a multidisciplinary team. Paterson and Mulligan [2] report on a South Australian survey where eight patients reported unauthorised disclosure of health information between health professionals, observing that

The difficulty lies in the fact that the doctor may take it for granted that a holistic approach to health care and any consequent information-sharing is in the best interests of patients whereas patients may neither expect nor approve of such an approach. [2]

## Integrated Care and Privacy

Modern evidence-informed medicine has moved toward integrated systems that include multidisciplinary teams, bio-psycho-social interventions, and shared follow-up care. Whilst the goal of this approach is to act in the best interest of patients, it can be viewed to be in tension with the principle of respect for patient autonomy [2]. Clinical integration requires the sharing of information between care providers. Some providers will be known to the patient while others (i.e. clinical supervisors, pathology and imaging services) will not, indeed some practitioners may not be considered members of the traditional health care team (i.e. school educational psychologists, disability support workers, and practitioners in the criminal justice sector) [3]. Effective integrated clinical care requires the sharing of clinical information across a team from a range of sectors and disciplines. The focus here will be on legislative provision for the sharing of health information within a multidisciplinary health care team.

## The Health Care Team in Legislation

Whilst a patient may broadly expect that health information will be shared within a “treating team”, including multidisciplinary health care providers, it is not always clear who is a health provider, as not all practitioners will be registered under National Law [4] and indeed may sit outside the “usual” understanding of a health professional.

New South Wales state health legislation provides a broad definition of both what constitutes a health service and who is a health provider. Definitions found in a number of health related legislation are based on the earlier the *Health Care Complaints Act 1993* (NSW) which was developed after extensive community consultation [5]. This defines a health practitioner as both registered and un-registered persons who provide health services. Additionally the definition of a “health service” is broad including public and private sectors, Aboriginal and Torres Strait Islander health practices and welfare services. Further, the *Health Records and Information Privacy Code of Practice 2005* (NSW) includes other human service agencies such as housing and education services [6]. Similarly, state Victorian Health legislation, in the *Health Complaints Act 2016* (Vic) defines a health service provider as a person who provides a health service [7]. The *Health Practitioner Regulation National Law (Victoria) Act 2009* defines a health service to include speech therapists, naturopaths, psychotherapists and support services necessary to implement any health services [8].

The Commonwealth defines a health service in the *Privacy Act 1988* (Cth) as a set of activities aimed at assessing, diagnosing, treating, maintaining, improving or managing health, disability and injury [9]. The Medical Benefits Scheme (MBS) regulations support this by including non-medical professionals including care coordinators such as social workers, Aboriginal health workers, education providers and probation officers [10]. Additionally, of relevance to the discussion here is the *Privacy Act 1988* (Cth) definition of an entity as an agency or organisation, and the definition of an “organisation” includes individuals, partnerships and any unincorporated association [9]. This concept of partnership or association is reinforced in the *Health Records and Information Privacy Act 2002* (NSW) which makes special exception for group practices by recognising their nature as a group of individuals providing a health service who by written agreement share premises, reception and combined or joint [health] records [11]. Such a partnership or unincorporated association is covered by the privacy provisions of the *Privacy Act 1988* (Cth).

It is clear from the above that a wide range of individuals and other “entities” can be considered health practitioners and health services for the purposes of privacy, health records, health complaints and health insurance legislation.

## Health Information Sharing

The Australian Federal Government addresses health privacy matters principally through the Australian Privacy Principles (APPs) within the *Privacy Act 1988* (Cth) [8,9] and are reflected in State legislation as Health Privacy Principles (HPPs). The general use and disclosure of health information (APP 6) permits organisations (public or private) to use and disclose health information for the purpose for which it was collected (primary purpose) and for other purposes that are related to the primary purpose and that are within the individual’s reasonable expectations (secondary purpose) (APP 6.2) [12]. If the information is sensitive the secondary purpose must be directly related to the primary purpose.

The NSW [11] and Vic [13] State legislation treatment of health information sharing mirrors the commonwealth law and notes

If information is collected in order to provide a health service to the individual, the disclosure of the information to provide a further heath service to the individual is a secondary purpose directly related to the primary purpose. [11]

This indicates that health information can be used or “shared” internally within a health care team, or disclosed or “shared” externally to a health care partner, provided the purpose is directly related to the original purpose it was collected, which might commonly be providing an effective health service.

## Conclusion And Recommendations

The definition of health provider, health organisation, health entity, health professional is broadly defined in privacy related legislation to cover most circumstances. Thus the definition of who might be a member of a health care team extends to all persons who provide a health service.

It is notable that within NSW clinical group practices who share premises and files are exempted from many of the health privacy principals as defined by legislation. With the recent developments of new interagency and public-private models of care, the question should be asked whether the intention of the legislators was to allow for exemption of similar broader health care and treatment teams, that operate in group practice such as multidisciplinary and integrated care teams.

Within NSW, HPPs make provision for clinicians to share information within the “health care” team, and with closely affiliated clinicians where there has been informed consent from the patient. It is also clear that where informed consent has not been obtained, health information can be used and disclosed (shared) for the purpose for which it was collected (the primary purpose) and for other purposes that are related to the primary purpose and that are within reasonable expectation.

It seems, therefore, that multidisciplinary interagency health care teams can collect and share health information, without direct consent, provided the purpose is closely related to the primary purpose it was collected. The holistic nature of health care is not fully appreciated by all, and therefore, the “sharing” of sensitive information with others in the “team” may not be clear to patients. It is, therefore, good practice to inform patients of the nature of the “multidisciplinary health care team” and advise them of the process of sharing health information among team members. There is, however, no legislative requirement to always have consent for the sharing of clinical information among members of the health care team, provided such sharing is consistent with both APPs and State HPPs. The sharing of health information between team members is an important component of the drive to improve the quality and safety of care.
